# Canine babesiosis in northern Portugal and molecular characterization of vector-borne co-infections

**DOI:** 10.1186/1756-3305-3-27

**Published:** 2010-04-08

**Authors:** Luís Cardoso, Yael Yisaschar-Mekuzas, Filipa T Rodrigues, Álvaro Costa, João Machado, Duarte Diz-Lopes, Gad Baneth

**Affiliations:** 1 Department of Veterinary Sciences, University of Trás-os-Montes e Alto Douro, Vila Real, Portugal; 2 Parasite Disease Group, Instituto de Biologia Molecular e Celular, Universidade do Porto, Portugal; 3 School of Veterinary Medicine, Hebrew University of Jerusalem, Rehovot, Israel; 4 Clínica Veterinária Dr. Duarte Diz-Lopes, Bragança, Portugal; 5 Clínica Veterinária Os Bichos, Chaves, Portugal

## Abstract

**Background:**

Protozoa and bacteria transmitted by arthropods, including ticks and phlebotomine sand flies, may cause a wide range of canine vector-borne diseases. Dogs can be simultaneously or sequentially infected with multiple pathogens. Canine babesiosis caused by *Babesia canis canis *and *Babesia canis vogeli *is known to occur in Portugal. This study assessed, by means of blood smear examination, PCR and DNA nucleotide sequencing, the presence of *Babesia *spp. and co-infecting agents *Leishmania*, *Anaplasma*/*Ehrlichia *and *Hepatozoon *in 45 dogs from northern Portugal clinically suspected of babesiosis.

**Results:**

Forty-four dogs (98%) had infection with *B. canis canis *and one with *B. canis vogeli*. Co-infections were detected in nine animals (20%). Eight dogs were found infected with two vector-borne agents: six with *B. canis canis *and *Leishmania infantum*; one with *B. canis canis *and *Ehrlichia canis*; and one with *B. canis canis *and *Hepatozoon canis*. Another dog was infected with three vector-borne pathogens: *B. canis vogeli*, *E. canis *and *L. infantum*. Overall, *L. infantum *was found in seven (16%), *E. canis *in two (4%), and *H. canis *in one (2%) out of the 45 dogs with babesiosis. Almost 90% of the 45 cases of canine babesiosis were diagnosed in the colder months of October (18%), November (27%), December (20%), February (13%) and March (9%). Co-infections were detected in February, March, April, May, October and November. Twenty-two (50%) out of 44 dogs infected with *B. canis *were found infested by ticks including *Dermacentor *spp., *Ixodes *spp. and *Rhipicephalus sanguineus*. Mortality (9%) included two co-infected dogs that died spontaneously and two with single infections that were euthanized.

**Conclusions:**

*Babesia canis canis *is the main etiological agent of canine babesiosis in northern Portugal. A higher sensitivity of *Babesia *spp. detection was obtained with PCR assays, compared to the observation of blood smears. Twenty percent of the dogs were co-infected with *L. infantum*, *E. canis *or *H. canis*. Furthermore, this is the first molecular identification of *H. canis *in dogs from northern Portugal.

## Background

A large variety of protozoa and bacteria transmitted by arthropods, including ixodid ticks and phlebotomine sand flies, may cause diseases in dogs and other vertebrate hosts [1,2]. Canine piroplasmosis is caused by several tick-borne *Babesia *and *Theileria *protozoal haemoparasites (termed piroplasms) and represents a pathological condition with significant veterinary medical importance in many parts of the world [3]. Mainly depending on the piroplasm species or subspecies, the effects of infection in dogs may range from a subclinical condition to severe and even fatal illness. Clinical abnormalities associated with piroplasmosis frequently comprise lethargy, anorexia, pale mucous membranes, icterus, hyperthermia, haemolytic anaemia, haemoglobinuria and thrombocytopenia [4,5]. Other factors, such as the canine host age and immunity [6], together with concomitant infections or diseases, also play a role in the potentially variable pathogenicity of the disease [7].

Morphological appearance in the erythrocytes and especially molecular analysis have allowed the differentiation of several large (3-5 μm) and small (0.5-2.5 μm) piroplasms of dogs [3]. In Europe, the causative agents of canine babesiosis include large *Babesia canis canis*, a subspecies transmitted by the tick *Dermacentor reticulatus *which causes a mild to severe disease, and *B. canis vogeli*, transmitted by *Rhipicephalus sanguineus *[8-11]. *Babesia canis vogeli*, the less virulent subspecies of *B. canis*, is also present in tropical and subtropical areas of Africa [12], Asia [13], Australia [14], North and South America [15,16]. The most virulent of the three subspecies, *B. canis rossi*, has been reported only in central and southern Africa [4,17]. Furthermore, another large yet unnamed *Babesia *sp., genetically related to *Babesia bigemina *of cattle, has been reported in dogs with clinical signs of babesiosis in North Carolina [18,19].

Small *Theileria annae*, a *Babesia microti*-like piroplasm [20] endemic in northwestern Spain [21], and *Babesia gibsoni *have also been detected as agents of babesial disease in European dogs [22-24]. Outside Europe, *T. annae *has been detected in one dog from Mississipi [25]. *Babesia gibsoni *has a wider distribution, with infections reported primarily from Asia [13] but also from America [15,26] and Australia [27]. One other genetically distinct small piroplasm species is recognised to cause canine babesiosis: *Babesia conradae *in southern California [28]. Furthermore, DNA of large *Babesia caballi *[24] and small *Theileria equi *[24,29] and *Theileria annulata *[30], in Europe, and an unnamed *Theileria *sp., in South Africa [31], have been detected in dogs, but their role in canine piroplasmosis needs to be confirmed.

The geographical distribution of canine piroplasms is largely determined by the ecological ranges of their vector arthropods [32]. Epidemiological surveillance of disease occurrence and prevalence is required to map local risk and forecast vector-borne infection scenarios. Canine babesiosis caused by large piroplasms is known to occur in northeastern Portugal [33], and both *B. canis canis *and *B. canis vogeli *have recently been identified in some naturally infected dogs from this area [34]. In areas where canine vector-borne diseases (CVBD) are endemic, dogs can be simultaneously or sequentially infected with more than one vector-borne agent, depending on the presence and abundance of arthropod vectors [1]. *Leishmania infantum *[35] and the rickettsiae *Anaplasma platys *and *Ehrlichia canis *are also proven agents of CVBD in northern Portugal [36]. The sand fly season runs from May to October in the Douro subregion of northern Portugal: *Phlebotomus perniciosus *and *Phlebotomus ariasi*, vectors of *Leishmania*, are most abundant in July and September, respectively [37]. Ticks of the species *D. reticulatus *and *R. sanguineus *have been found to infest dogs in northeastern Portugal [38-40]. In addition to transmitting *B. canis vogeli *and *E. canis*, and presumably *A. platys*, *R. sanguineus *is also the main vector for protozoan *Hepatozoon canis *in Europe [41,42]. This study assessed the presence of *Babesia *spp. and the co-infecting pathogens *Leishmania*, *Anaplasma*/*Ehrlichia *and *Hepatozoon *in dogs from northern Portugal clinically suspected of babesiosis by means of blood smear examination, PCR and DNA nucleotide sequencing.

## Methods

### Animals and samples

Forty-five dogs from Alto Trás-os-Montes and Douro (northern Portugal) suspected of babesiosis, between October 2007 and March 2009, were included in this study. The two subregions of Alto Trás-os-Montes (north) and Douro (south) cover a total area of 12,282 sq. km and are bordered by Spain to the north and east. The terrain is hilly, and agriculture is the main source of occupation and income. No history of travel to southern Portugal or to Spain was obtained for any of the 45 dogs. After recording the dog's signalment, each animal was physically examined and blood samples were collected from the ear tip to prepare glass slide smears and to assess microhaematocrit (HCT). The thin smears were air-dried, fixed with methanol, Giemsa-stained and then examined under light microscopy (magnification 1000×; 100 fields) for detection of babesial piroplasms and other possible infective agents. Additional samples of peripheral or venous blood were spotted onto individual filter papers (7.5 cm × 2.5 cm; GB 002 Schleicher and Schuell, Dassel, Germany) allowed to air-dry and stored at -20°C until further use. Dogs were also examined for the presence of infesting ticks. The anti-babesial treatment administered was recorded and the disease outcome followed.

### DNA extraction

Filter paper portions, corresponding to approximately 20 μl of spotted blood, were cut out by use of individual sterile scalpel blades and put into sterile tubes for DNA extraction [43]. DNA was extracted by adding 300 μl of lysis buffer [50 mM NaCl, 50 mM Tris, 10 mM EDTA (pH 8.0)], proteinase K to a final concentration of 250 μg/ml and Triton X-100 (20%) to a final concentration of 1%. Following a 2 h incubation at 56°C and an inactivation of proteinase K at 90°C for 10 min, 300 μl of phenol (75%), chloroform (24%) and isoamylalcohol (1%) mixture were added, vortexed and centrifuged (12,000 × *g*) for 4 min. The supernatant was collected and 300 μl of a mixture of phenol (50%), chloroform (48%) and isoamylalcohol (2%) were added, vortexed and centrifuged (12,000 × *g*) for 4 min. The supernatant was collected and 300 μl of a mixture of chloroform (96%) and isoamylalcohol (4%) were added, vortexed and centrifuged (12,000 × *g*) for 4 min. The supernatant was collected, and 1:10 volume of Na-acetate (3 M) and one volume of ice cold 100% isopropanol (-20°C) were added and incubated over night at -20°C. Following centrifugation (14,000 × *g*) at 4°C for 30 min, the supernatant was discarded and the pellet was washed with 150 μl of ethanol (75%, -20°C) and centrifuged (13,000 × *g *4°C) for 15 min. The supernatant was discarded and the pellet was air-dried. The DNA was then hydrated with 30 μl of double-distilled H_2_O for 1 h at 50°C.

### PCR assays for Babesia, Anaplasma/Ehrlichia, Hepatozoon and Leishmania

Primers PIRO-A and PIRO-B (Table 1) were used to amplify an approximate 408 bp fragment of the 18S ribosomal RNA (rRNA) gene of *Babesia *spp. by PCR [44]. Amplification was done under the following conditions: 94°C for 1 min followed by 39 cycles of 94°C for 45 sec, 62°C for 45 sec and 72°C for 45 sec.

**Table 1:** Primer sets for the PCR amplification and sequencing of vector-borne infective agents used in the study

Agent	Primers	Reference(s)
*Babesia *spp.	PIRO-A: 5'-AAT ACC CAA TCC TGA CAC AGG G-3' PIRO-B: 5'-TTA AAT ACG AAT GCC CCC AAC-3'	[44]

*Anaplasma *spp./*Ehrlichia *spp.	EHR16SD: 5'-GGT ACC YAC AGA AGA AGT CC-3' EHR16SR: 5'-TAG CAC TCA TCG TTT ACA GC-3'	[45,46]

*Hepatozoon *spp.	HEP-F: 5'-ATA CAT GAG CAA AAT CTC AAC-3' HEP-R: 5'-CTT ATT ATT CCA TGC TGC AG-3'	[47,48]

*Leishmania *spp.	ITS-219F: 5'-AGC TGG ATC ATT TTC CGA TG-3' ITS-219R: 5'-ATC GCG ACA CGT TAT GTG AG-3'	[49]

Primers EHR16SD and EHR16SR (Table 1) were used to amplify an approximate 345 bp fragment of the *Ehrlichia *and *Anaplasma *genera 16S rRNA gene [45]. PCR amplification was performed under the following conditions: 95°C for 5 min; 40 cycles of 94°C for 30 sec, 55°C for 30 sec and 72°C for 90 sec; then final extension at 72°C for 5 min [46].

PCR for the detection of *Hepatozoon *was performed using primers (125 nM each) HEP-F and HEP-R [47,48] (Table 1). The following conditions were used to amplify a partial 666 bp fragment of the 18S rRNA gene sequence of *Hepatozoon *spp.: 95°C for 5 min; 35 cycles of 95°C for 20 sec, 57°C for 30 sec and 72°C for 90 sec; and 72°C for 5 min. PCR was performed using Syntezza PCR-Ready High Specificity (Syntezza Bioscience, Israel).

A 265 bp fragment within the internal transcribed spacer 1 (ITS1) region of the *L. infantum *rRNA operon was amplified by real-time PCR using the primers ITS-219F and ITS-219R (Table 1) and then evaluated by high resolution melt (HRM) analysis [49]. The PCR reaction was performed in a total volume of 20 μl containing 5 μl DNA, 40 nM of each primer, 10 μl Thermo-start PCR Master Mix (Thermo-start ABgene, Rochester, NY, USA), 0.6 μl 100-fold diluted SYTO9 (Invitrogen, Carlsbad, CA, USA), and sterile, DNase/RNase-free water (Sigma, St. Louis, MO, USA) using a Rotor-Gene 6000 real-time PCR machine (Corbett Life Science). Initial denaturation for 15 min at 95°C was followed by 40 cycles of denaturation at 5 sec at 95°C per cycle, annealing and extension for 30 sec at 57°C, and final extension for 1 sec at 76°C. This was followed by a conventional melting step from 60°C to 95°C at 1°C/sec, after which the temperature was slowly decreased from 90 to 50°C (1°C/sec) to allow re-annealing. In the final step, HRM analysis was carried out increasing the temperature from 75°C to 90°C at 0.4°C/sec increments [49].

Positive *B. canis*, *E. canis*, *H. canis *and *L. infantum *DNA control samples from the blood of naturally infected dogs negative by PCR for other pathogens, and negative DNA controls from colony-bred dogs negative by PCR for vector-borne pathogens were run with each corresponding PCR reaction.

### Sequencing

DNA sequencing was performed at the Center for Genomics Technologies, Hebrew University of Jerusalem. Obtained DNA sequences were evaluated with ChromasPro software version 1.33 and compared for similarity to sequences in GenBank, using the BLAST program hosted by NCBI, National Institutes of Health, USA http://www.ncbi.nlm.nih.gov.

### Statistical analysis

The Chi-squared or Fisher's exact tests were used to compare proportions. Differences between independent groups were analyzed with the Mann-Whitney U test [50]. Analyses were performed with SPSS 10.0 software for Windows, with a probability (*p*) value < 0.05 as statistically significant.

## Results

The 45 dogs suspected of having babesiosis consisted of 24 males and 21 females. Age was not determined for seven dogs; in the remaining 38 animals it ranged from 2 months to 14 years (median value of 3.0 years [interquartile range: 1.1-4.8]). There were 28 dogs from nine defined breeds and 16 mongrels; breed was not determined for one dog. The most represented breeds were Podengo (n = 17) and the Brittany (n = 3).

The clinical signs found on physical examination in the 38 dogs were: lethargy (n = 24; 63%), red urine (n = 19; 50%), hyperthermia (n = 18; 47%), anorexia (n = 17; 45%), pale mucous membranes (n = 17; 45%), hypothermia (n = 9; 24%), yellow mucous membranes (n = 5; 13%), vomiting (n = 4; 11%), abdominal pain (n = 3; 8%), ataxia (n = 2; 5%), uterine discharge (n = 2; 5%), cough (n = 1; 3%), gingival petechiae (n = 1; 3%) and ocular discharge (n = 1, 3%). Blood tests showed that 26 (79%) out of 33 dogs were anaemic, with a HCT value below the reference interval (37-55%). Data on physical examination were not determined in six and HCT was not evaluated in 12 out of the 45 dogs. Peripheral blood smear evaluation showed intraerythrocytic piroplasms morphologically compatible with *B. canis *(3-5 μm long and mainly occurring in pairs or single ring shapes) in 41 (91%) of the 45 clinically suspected dogs. Presence of other infective agents could not be confirmed by microscopy of blood smears. Those 41 dogs and the remaining four suspected animals were all found positive for *Babesia *spp. by PCR. Further sequence analysis revealed that 44 dogs (98%) were infected with *B. canis canis *(98-100% relatedness to the GenBank closest sequence) and one with *B. canis vogeli *(100% relatedness).

Results concerning the observation of large babesial parasites in smears, and PCR amplification with sequencing of *Babesia *and co-infecting *Anaplasma*/*Ehrlichia*, *Hepatozoon *or *Leishmania *agents are shown in Table 2. Thirty-six dogs were found infected only with *B. canis canis*, whereas co-infections were detected in nine dogs (20%). Eight dogs were found infected with two vector-borne agents: six dogs with *B. canis canis *and *L. infantum*; one dog with *B. canis canis *and *E. canis*; and one dog with *B. canis canis *and *H. canis*. Another dog was found infected with three vector-borne organisms: *B. canis vogeli*, *E. canis *and *L. infantum*. Overall, *L. infantum *was found in seven dogs (16%), *E. canis *in two dogs (4%), and *H. canis *was found in one (2%) out of the 45 dogs diagnosed with babesiosis.

**Table 2:** Comparison of results from blood smear examination, PCR and sequence analysis in 45 dogs suspected of babesiosis.

Blood smear	PCR and sequencing		Dogs (n)
		
	*Babesia *spp.	Co-infection	
Large piroplasms	*B. canis canis *(98-100%)	Negative	32
	*B. canis canis *(99-100%)	*L. infantum *(99-100%)	6
	*B. canis canis *(99%)	*E. canis *(100%)	1
		*H. canis *(99%)	1
	*B. canis vogeli *(99%)	*E. canis *(100%) and *L. infantum *(99%)	1

Piroplasms not found	*B. canis canis *(100%)	Negative	4

Percentage values refer to species or subspecies relatedness to the GenBank closest sequences.

**Figure 1:**
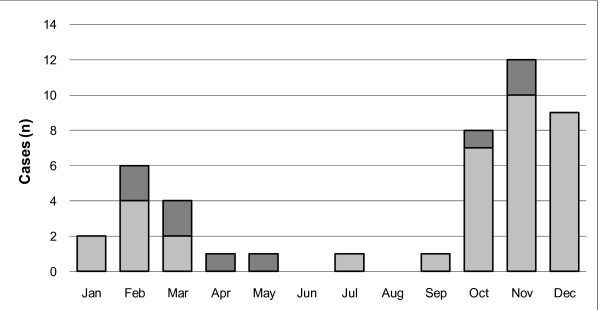
**Monthly distribution of canine babesiosis and of co-infection cases in 45 dogs from northeastern Portugal**. Light grey bars represent 36 cases of babesiosis with no co-infecting agents (*Anaplasma*/*Ehrlichia*, *Hepatozoon *or *Leishmania*). All 36 dogs infected solely with *Babesia *had *B. canis canis *infection. Dark grey bars represent nine cases of babesiosis with one or two concurrent infecting agents along with *B. canis canis *(n = 8) or *B. canis vogeli *(n = 1), respectively.

Monthly distribution of the 45 observed cases of canine babesiosis was as follows: January (n = 2), February (n = 6), March (n = 4), April (n = 1), May (n = 1), July (n = 1), September (n = 1), October (n = 8), November (n = 12) and December (n = 9). No cases were observed during June or August. Co-infection cases were detected in February, March, April, May, October and November (Figure 1 and Table 3).

**Table 3:** Signalment, clinical signs and vector-borne agents in nine co-infected dogs.

Agents	Gender	Breed	Age (months)	Clinical signs	HCT (%)	Ticks	Month	Clinical outcome (imidocarb treatment)
*B. canis canis *and *L. infantum*	F	Dalmatian	72	ND	ND	No	October	Recovered
	M	Mongrel	02	Hyperthermia, PMM, RU	20	Yes	November	Died
	M	Mongrel	02	ND	10	Yes	November	Recovered
	F	Mongrel	36	ND	40	Yes	February	Recovered
	M	Mongrel	36	Hyperthermia, RU	40	Yes	February	Recovered
	M	Mongrel	ND	PMM, RU	15	Yes	March	Recovered

*B. canis canis *and *H. canis*	F	Podengo	47	Hyperthermia, PMM, RU	25	Yes	May	Recovered

*B. canis canis *and *E. canis*	M	German pointer	78	Anorexia, hyperthermia, lethargy, RU	40	No	March	Recovered

*B. canis vogeli*, *E. canis *and *L. infantum*	M	Podengo	36	Anorexia, hypothermia, lethargy, YMM	03	Yes*	April	Died

F: female; HCT: haematocrit (normal range: 37-55%); M: male; ND: not determined; PMM: pale mucous membranes; RU: red urine; YMM: yellow mucous membranes. *R. sanguineus.

Ticks were detected on 22 dogs and tick identification was performed in 10 of these animals. *Dermacentor *spp. found on four dogs infected with *B. canis canis*, and *Ixodes *spp. on four other dogs also infected with *B. canis canis*. *Rhipicephalus sanguineus *was present on the dog found co-infected with *B. canis vogeli*, *E. canis *and *L. infantum*. Another dog was co-infested with *Dermacentor *spp. and *R. sanguineus *and infected with *B. canis canis*. Ticks were not detected on 22 dogs and information from one animal was not determined. Twenty-two (50%) out of 44 dogs infected with *B. canis *were found infested by ticks; and seven (78%) out of nine dogs with co-infections had ticks.

Table 3 provides information on gender, breed, age, clinical signs, HCT, detected vector-borne agents, presence of ticks and month of sampling for the nine dogs found co-infected. Differences between HCT values in a group of eight co-infected (*B. canis canis *and *L. infantum*, *E. canis *or *H. canis*) and another group of 25 dogs not found co-infected (infected solely with *B. canis canis*) were not statistically significant (Mann-Whitney U test [MWU]; *p *= 0.449); the differences in HCT between a group of five dogs co-infected with *B. canis canis *and *L. infantum *and those 25 dogs not found co-infected were also not significant (MWU; *p *= 0.504). The same was true for age differences between eight co-infected and 30 dogs not found co-infected (MWU; *p *= 0.971); and for differences between age in five dogs co-infected with *B. canis canis *and *L. infantum *and in those 30 dogs solely infected with *B. canis canis *(MWU; *p *= 0.409). No significant difference, although close to significance, was observed when comparing the proportions of co-infected mongrel dogs (6/16; 37.5%) and that of co-infected defined breed animals (3/28; 10.7%) (Fisher's exact test; *p *= 0.053). Statistically significant differences were not found upon comparison of proportions of co-infected male (6/24; 25%) and co-infected female dogs (3/21; 14.3%); or co-infected dogs among those infested with ticks (7/22; 31.6%) and co-infected dogs among those with no ticks (2/22; 9.1%).

Of the 45 dogs diagnosed with babesiosis, four (9%) died. Despite treatment with imidocarb dipropionate, two dogs died (22%) out of the nine found co-infected (Table 3). One of these animals was found infected with *B. canis vogeli*, *E. canis *and *L. infantum*; and the other one with *B. canis canis *and *L. infantum*. Two other dogs with babesiosis were subject to euthanasia as requested by their owners. Molecular analysis later revealed infection with *B. canis canis *in these two animals. Forty-one dogs - including seven co-infected with *B. canis canis *and *L. infantum*, *E. canis *or *H. canis *and 34 with single infection (only *B. canis canis*) - clinically recovered after treatment with imidocarb dipropionate.

## Discussion

In this study, babesiosis in northern Portugal was found to be caused predominantly by infection with *B. canis canis*, with *L. infantum *as the most prevalent co-infecting agent. Although ticks were found only on approximately half the dogs with babesiosis, a considerable proportion of the co-infected dogs were infested by ticks (78%). In addition, canine babesiosis was diagnosed mainly from October to March, when climate conditions favour the activity of *Dermacentor *spp. ticks [38]. In agreement with previous studies [34,36], molecular confirmation of the presence of vector-borne pathogens in northern Portugal has been reestablished for *B. canis vogeli *and *E. canis*. In addition, to our best knowledge, this is the first report of molecular identification of *H. canis *in dogs from northern Portugal. Based on the observation of *H. canis *gamonts in neutrophils, it had been previously assumed that *H. canis *is the species involved in canine infection, but genetic characterization was not available at the species level [40].

*Babesia canis canis *was detected in 98% of the 45 cases of canine babesiosis. This could be due to a higher prevalence of infected dogs or tick vectors in the study area, in comparison to *B. canis vogeli*, or to its more virulent nature. Due to the severity of clinical presentation, as compared with the relatively milder signs induced by *B. canis vogeli *[7], dogs infected with *B. canis canis *would potentially be brought in more often for veterinary consultation [34]. No comparisons were done between the dogs found infected with each one of the two subspecies of *B. canis*, because there was only one animal found infected with *B. canis vogeli*. An investigation of 164 Italian dogs suspected of tick-borne disease found *B. canis canis *in 34 and *B. canis vogeli *in 11 different cases [51]. This same study showed that clinical cases with *B. canis vogeli *infection did not present a homogenous clinicopathological pattern as observed in the clinical cases of infection with *B. canis canis*. Furthermore, in these dogs from Italy, *B. canis vogeli *infections were found in three puppies (1-2 months) associated with severe haemolytic anaemia (fatal disease in one case) but with no reported concomitant disease; in one other young dog with chronic renal failure; and in four older dogs with leishmaniosis (n = 1), immunosuppression (n = 2) or post splenectomy (n = 1) [51].

In the present study, co-infection with *L. infantum *was more prevalent (16%) than with *E. canis *(4%) or *H. canis *(2%) among the 45 dogs with babesiosis. Due to relatively lower parasite loads of *Leishmania *in the blood, compared with other tissues, use of blood to assess infection with *Leishmania *may have limited the sensitivity of detection; however, the use of highly sensitive quantitative real time PCR for *Leishmania *spp. in this study probably improved the prospects of detection, when compared with conventional PCR assays [49,52]. In the present study, large babesial piroplasms were detected in blood smears of nearly 90% of the clinically suspected dogs further confirmed as infected with *B. canis canis *or *B. canis vogeli*. Parasites were not detected in the smears of four dogs found infected with *B. canis canis *and diagnosed by PCR and sequencing. Microscopy may lack sensitivity in dogs clinically suspected of babesiosis, possibly due to low parasitaemia [2,7].

The arthropods described as vectors of the detected pathogens - *D. reticulatus *for *B. canis canis*; *Phlebotomus *spp. for *L. infantum*; and *R. sanguineus *for *B. canis vogeli*, *E. canis*, and *H. canis *- are present in northern Portugal [37,39]. In this study, *Dermacentor *spp. were found on dogs infected with *B. canis canis *and *R. sanguineus *on one dog co-infected with *B. canis vogeli *and *E. canis *(and also *L. infantum*). History of travel outside this area, where canine leishmaniosis and babesiosis are endemic, was not obtained for any of the dogs. This situation supports the assumption that infections with *Babesia*, *Leishmania *and the other vector-borne agents were acquired locally.

The only dog found infected with *B. canis vogeli *in our study also had co-infection with *E. canis *and *L. infantum*. It is possible that chronic subclinical or acute infection with *B. canis vogeli *had been made clinically apparent by these co-infections. We had previously detected one clinical case in a dog from northern Portugal infected with *B. canis vogeli *concurrently with *A. platys *[36]. *Babesia canis vogeli *and *E. canis *share the same vector species, i.e. *R. sanguineus *ticks. The co-infected dog may have been exposed to arthropods infected with single pathogen species at different points in time or to vector(s) concurrently infected with multiple agents [1]. Co-infections with *Leishmania *and tick-borne organisms may affect the severity of CVBD and the variety of associated clinical signs [53]. In a study with beagle dogs naturally exposed to *E. canis *and *L. infantum*, the frequency of clinical signs (lymphadenomegaly, splenomegaly, epistaxis, onychogryposis, dermatits and weight loss) was significantly different between animals with dual infection and those with single infection [54]. However, the clinical signs of co-infections with two or more vector-borne organisms are often difficult to be specifically assigned to each one of the infecting agents [55]. In the present study, although a complete clinicopathological evaluation was not performed, especially blood cell counts, no significant differences among HCT values were found between the co-infected dogs and those with one single infection detected. Nevertheless, dogs with co-infections had a lower survival rate when compared to those with single infection. In fact, two dogs (22%) died out of the nine found co-infected: one with *B. canis vogeli*, *E. canis *and *L. infantum*, and the other one with *B. canis canis *and *L. infantum *infection. From the 36 dogs found infected only with *B. canis canis*, two (6%) were euthanized and the remaining 34 animals (94%) clinically recovered with the anti-babesial treatment.

Another study in rural and hunting dogs (n = 473), from northeastern Portugal, showed a 15% seroprevalence of antibodies to *E. canis*, and a 2% prevalence of *Hepatozoon *spp. in blood smears [40]. Six dogs were simultaneously found to be seropositive for *E. canis *and positive for *Hepatozoon *spp., but PCR did not detect *Ehrlichia *or *Anaplasma *in any of those animals. Nevertheless, *E. canis *DNA was sequenced from four other dogs, thus revealing a 0.9% prevalence of infection. No babesial piroplasms were found in blood smears from all the dogs included in the same study. The differences between these prevalence rates for *E. canis*, as detected by molecular methods, and piroplasms and those observed in the present study may be explained by a different sample population and the methods used. In fact, only 10% of the dogs studied by Figueiredo [40] were clinically suspected of bacterial or protozoal diseases, and infection with *Babesia *spp. was not assessed molecularly by this author, whereas all the dogs in the present study were positive to *Babesia *spp. and thus exposed to at least one species of tick-borne pathogen.

In this study, two littermates aged two months old were both found co-infected with *B. canis canis *and *L. infantum*. This finding could suggest the possibility of transplacental transmission of *L. infantum *[56] and/or *B. canis canis *[57]. However, both puppies were found infested with ticks (species not identified), which should be regarded as the most likely source of transmitting *Babesia *to them. Regarding infection with *Leishmania*, these animals were born in early October and transmission by phlebotomine sand flies should still be considered [37]. Data on physical examination were not available for one of the dogs. The other dog presented hyperthermia, pale mucous membranes and red urine, which could be attributed to *B. canis canis *infection. Both animals suffered from anaemia, and one of the dogs died. It is not clear whether infection with *L. infantum *contributed to the clinical abnormalities in these two puppies and whether they were suffering from pathological effects of infection with *Leishmania*. Other tests, including serological analysis for antibodies to *Leishmania*, complete blood count, serum biochemistry panel and urinalysis, could have been helpful in clarifying the clinical status of these two and of the other seven co-infected dogs as well [58]. In general, the incubation period of canine babesiosis is short (4-21 days) [3], while the incubation of canine leishmaniosis is much longer (2 months to several years) [59].

The trend of canine babesiosis seasonality found in the present study is further strengthened by results from an additional study (Diz-Lopes D, Rodrigues FT: Babesiose canina - estudo clínico no Nordeste Transmontano [unpublished abstract]. V Congresso Veterinário Montenegro: 17-18 January 2009; Porto). A higher occurrence of disease was found during October and November (21 cases during each month) in 98 dogs from northeastern Portugal diagnosed with babesiosis by clinical examination and by observation of intraerythrocytic large piroplasms, from January 2005 to December 2008. Considerable numbers of canine babesiosis cases were also found from December to May, with monthly values ranging between 6% and 11%. Sixty-two per cent of all the cases were detected in hunting dogs, and 52% of all the affected animals were Podengo dogs (Diz-Lopes D, Rodrigues FT: Babesiose canina - estudo clínico no Nordeste Transmontano [unpublished abstract]. V Congresso Veterinário Montenegro: 17-18 January 2009; Porto). In the present study, it was found that approximately 90% of the 45 cases of babesiosis in dogs from northern Portugal were diagnosed in October (18%), November (27%), December (20%), February (13%) and March (9%), i.e. autumn and winter months. In central Europe, the occurrence of canine babesiosis due to *B. canis *has been found to change in an annual seasonal pattern, although exact time of beginning and ending of *Dermacentor *spp. activity is strongly correlated with specific local climate conditions [60]. In fact, epidemiological and clinical surveillance studies are needed for mapping the risk of babesiosis and other CVBD in different geographical regions.

A study in urban and rural dogs (n = 651) from Hungary revealed a 6% seropositivity to *B. canis *[61]. Seroprevalence to *B. canis *was significantly different for German shepherd and Komondor dogs, suggesting a genetic predisposition to chronic subclinical infection (carrier state) with long-term maintenance of seropositivity. A higher prevalence of specific antibodies in three out of four Komondors, a local breed, was explained by an increased risk of them having unnoticed ticks attached to their heavy hair coat [61]. In the present study, *B. canis canis *was found in males and females, younger and older dogs, from nine defined breeds and particularly from mongrels. There was no clear distinction of age and sex between single-infected and co-infected dogs. When comparing the proportions of co-infected mongrel dogs (~38%) and that of co-infected defined breed animals (~11%), there was a quantitative but not significant difference. Mongrels, Podengo and Brittany dogs represented the larger part of those found affected by babesiosis. Rather than a genetic or breed predisposition, this situation probably reflects the fact that these dog breeds and crosses are popular and over-represented in northern Portugal. Furthermore, a considerable percentage of these dogs live outdoors and are used for hunting activities in the field, where they face a higher risk of contacting with infected arthropod vectors.

*Theleria annae *may cause severe illness in dogs, including renal failure, and is endemic in northwestern Spain [21], which borders part of the area where the present study was carried out. To our knowledge, there are no written reports of autochthonous canine *T. annae *infection in Portugal. Nevertheless, due to the increasing mobility of dogs and the existence of competent or presumptive vectors, piroplasms may spread into non-endemic areas [1,2].

## Conclusions

In conclusion, this study confirmed the presence of *B. canis canis *and *B. canis vogeli *as agents of babesiosis in dogs from northern Portugal, with a large majority of the clinical cases found with the former piroplasm. A higher sensitivity of *Babesia *spp. detection was obtained by use of PCR assays, compared to microscopy of blood smears. Co-infections with some other vector-borne agents were also detected and molecularly characterized, namely *L. infantum*, *E. canis *and *H. canis*. Detection and identification of species and subspecies of pathogens, either in single or in co-infection, are necessary for the treatment, clinical management and prevention of CVBD.

## Competing interests

The authors declare that they have no competing interests.

## Authors' contributions

Conceived and design the study: LC, YYM and GB. Collected and characterized clinical samples: FTR, AC, JM and DDL. Performed PCR and genetic analysis: YYM. Analyzed data, drafted and revised the manuscript: LC and GB. All authors gave final approval of the version to be submitted.
